# Temporal sequence learning via adaptation in biologically plausible spiking neural networks

**DOI:** 10.1186/1471-2202-15-S1-P85

**Published:** 2014-07-21

**Authors:** Renato Duarte, Peggy Seriès, Abigail Morrison

**Affiliations:** 1 Bernstein Center Freiburg, Albert-Ludwig University of Freiburg, Freiburg im Breisgau, 79104, Germany; 2 Institute for Adaptive and Neural Computation, School of Informatics, University of Edinburgh, Edinburgh, EH8 9AB, UK; 3 Institute of Neuroscience and Medicine (INM-6), Computational and Systems Neuroscience, Jülich Research Center, Jülich, 52425, Germany; 4 Institute of Cognitive Neuroscience, Faculty of Psychology, Ruhr-University Bochum, Bochum, 44801, Germany

## 

Ecologically relevant computations are carried out by a complex interaction of adaptive dynamics, through a variety of activity-dependent modifications of synaptic and intrinsic neuronal properties. Such modifications ought to be robust and reliable enough to endow neuronal circuits with the ability to learn from and operate upon complex, dynamic environmental variables.

On the lower levels of the cortical processing hierarchy, continuous data streams representing the environment are parsed, in order to isolate and attend to salient and invariant features ('perceptual objects'), upon which higher order cortical networks will operate, by flexibly evaluating the dynamic relations between such structural elements [1]. The formation of stable representations of spatial/spectral environmental features (stimulus selectivity) along with the related ability to discriminate such features and their combinations is known to be continuously shaped and refined by synaptic plasticity mechanisms, and it has been recently demonstrated that correlation-based inhibitory plasticity has an important role to play in such computations (see, for example, [2]). However, in order to adequately process information, neural circuits must not only develop stable internal representations of perceptual objects, but also reflect and represent the continuous unfolding structure of its input, which is poised with intricate temporal dependencies. Much less is currently known about the acquisition of complex temporal relations between stimuli and the (possibly specialized) role played by different adaptation mechanisms involved in this process.

In this work, we study the properties of biologically realistic networks of LIF neurons, with differentially modulated, dynamic excitation and inhibition, combining well established as well as more recent phenomenological models of synaptic plasticity [2,3]. Explicitly embedded or entirely self-organized, input-specific neuronal assemblies are driven by stimulus sequences that contain complex temporal dependencies and signal propagation throughout these assemblies is gated by transient disruptions of E/I balance, in order to 'prime' the network to learn the underlying transitional probabilities and input statistics through targeted modifications of these 'gating' synapses. We explore the representational properties developed by these networks and the impact of the different plasticity rules in shaping the network's learning abilities while maintaining stable global dynamics. Furthermore, we assess the network's ability to extract complex temporal dependency rules between sequence elements and to use the acquired knowledge to make predictions about upcoming sequence elements.
